# A bibliometric analysis of the 50 most cited articles about artificial intelligence in electrocardiogram

**DOI:** 10.1186/s43044-025-00647-x

**Published:** 2025-05-29

**Authors:** Muhammad Arslan Ul Hassan, Sana Mushtaq, Abdul Rehman, Mohammed Abdulkarem Al-Qaisi, Zhen Yang

**Affiliations:** 1 Ningxia Medical University, Yinchuan, China; 2 General Hospital of Ningxia Medical University, Yinchuan, China

**Keywords:** Artificial intelligence, Electrocardiogram, ECG, Bibliometric analysis

## Abstract

**Background:**

Artificial intelligence (AI) is a modern tool that increases the diagnostic precision of the classical electrocardiogram (ECG). The objective of this bibliometric analysis was to identify the 50 most cited articles in the domain of AI in ECG, emphasizing publication trends, citation metrics, prominent authors and journals, leading institutions, and significant contributing countries.

**Results:**

The 50 most cited articles on AI in ECG were published between 2000 and 2020 across 25 journals. The mean citations per article were 488.0, with the highest citations count being 1870. ‘IEEE Transactions on Biomedical Engineering’ and ‘Computers in Biology and Medicine’ published the highest number of articles, while Rajendra Acharya U and RS Tan were the most contributing authors. The USA and China had a total of 14 publications, and Singapore was the country with most collaborations.

**Conclusions:**

This bibliometric analysis provides clinicians and researchers with an overview of evolution and progression of AI in the domain of ECG. Improved collaborations among different countries and institutions are essential for achieving advancements in the utilization of AI in ECG.

## Background

Artificial intelligence (AI) has grown into a revolutionary tool for modern healthcare system, transforming diagnostic accuracy and precision, clinical decision-making, and patient-centered management [1]. AI improves the interpretation of complicated medical data by using machine learning algorithms and neural networks, facilitating precise diagnosis of a variety of diseases [2, 3]. Cardiovascular disorders rank among the top three domains that researchers have focused on in the growing body of literature on AI [4]. Application of AI in cardiovascular domain has gained significant attention in the past, especially in the field of electrocardiogram (ECG) [5].

Since Willem Einthoven's pioneering development of the ECG in the late nineteenth century, cardiovascular system has been profoundly shaped by technological innovation [6]. ECG is a diagnostic technique that entails detecting and measuring electrical activity of the heart, which is subsequently transcribed into a graphical format for analysis in the clinics [7]. ECG serves as a critical tool in clinical practice, not only for identifying conduction abnormalities but also for assessing a wide range of cardiac pathologies, thereby supporting timely diagnosis and patient management. Its versatility in detecting both structural and functional heart irregularities underscores its indispensable role in improving cardiovascular care outcomes [8].

Today, AI emerges as an evolution of Einthoven's legacy, enhancing the ECG's diagnostic capabilities while offering clinicians nuanced insights to improve patient outcomes, streamline workflows, and address complex cardiac challenges with greater precision [9]. Moreover, AI in ECG has also demonstrated promise in risk stratification and patient prognosis prediction, supporting individualized and patient-centered treatment plans [10].

Apart from these advances in the literature, a thorough bibliometric analysis assessing the research patterns and trends, citation analysis, networks of collaboration between authors and countries, and thematic development of AI in ECG is still lacking. Although individual studies have examined and reported about AI usage in ECG, a comprehensive evaluation of worldwide research output and highlighting the significant contributions is still a need. This type of analysis can help in determining the knowledge gaps, directing future research, and maximizing the integration of AI usage in ECG and clinical cardiology.

The aim of this bibliometric analysis is to bridge this gap by systematically analyzing the top 50 most cited articles on AI in ECG. This bibliometric analysis of the 50 most cited articles seeks to delineate this field's conceptual boundaries by analyzing the publication timeline and trends, authorship and institution networks, keyword clusters, and citation dynamics. Moreover, the findings of this analysis will help the researchers, clinicians, and policymakers make informed decisions regarding AI-driven innovations in the field of cardiovascular medicine.

## Method

Approval from any ethics committee or the institutional review board was exempted for this bibliometric study as the data utilized for it were available publicly.

### Data source

Science Citation Index Expanded database from the Web of Science Core Collection (WoSCC) was utilized to acquire and extract the data for this study. Web of science is one of the most extensive and comprehensive databases available, providing substantially greater journal coverage and assessments of journal quality [11]. Moreover, the majority of bibliometric studies have employed this database to extract data [12].

### Search strategy

The search query on WoSCC utilized an advanced mode with topic search, along with the keyword strategy comprising of [TS = (Artificial Intelligence) OR (AI) OR (Machine Learning) OR (Neural Network) OR (Deep Learning) OR (Computational Intelligence) OR (Computer Reasoning) AND (Electrocardiogram) OR (ECG) OR (EKG) OR (Electrocardiography)]. This search strategy produced 87,731 articles, which were further limited to those written exclusively in English language, yielding 84,729 articles. Furthermore, the search was restricted to original articles only, yielding a total of 59,523 articles. Subsequently, the articles were organized by the number of citations, starting with the highest. The date range spanned from the database's inception till March 2025.

### Inclusion and exclusion criteria

We carefully reviewed the articles directly on the Web of Science website, assessing their relevance based on the title, abstract, keywords, and the full text when necessary. Only those studies that included both the primary keywords, AI and ECG, were selected. Additionally, we also included studies that described any method used for identifying, classifying, or interpreting ECG through AI.

Conversely, articles that did not focus on AI in ECG were excluded. We also excluded studies related to human or biometric identification through ECG, as well as research involving smartwatches and other wearable devices. Furthermore, review articles, abstracts, guidelines, consensus statements, scientific statements, systematic reviews, and meta-analyses were not included in this study.

### Data extraction and analysis

Two authors (MAUH and SM) independently carried out all the steps, ensuring an unbiased selection process of articles. Any disagreements regarding article selection were resolved through discussion or, when needed, by consulting the senior author (ZY). Both authors completed the search independently within a single day on March 19, 2025. Out of the final 59,523 search results, the top 474 were assessed, and 50 articles met the inclusion criteria. The full manuscripts of these selected articles were then obtained and thoroughly reviewed to extract relevant data.

The level of evidence (LOE) was evaluated for all the included articles to assess the relative risk of bias. Both authors independently applied the Oxford Centre for Evidence-Based Medicine guidelines to determine the LOE. Additionally, the citation density of each article, reflecting its impact over time, was calculated by dividing the total number of citations till the date of analysis with the number of years since the publication of the article as done in previous literature. For data analysis, we utilized R program (version 4.4.1), VOSviewer (version 1.6.20), and Statistical package for the social sciences (SPSS for Windows, version 27, IBM corp., Armonk, NY, USA). The R program was used along with the bibliometrix package, integrating"biblioshiny"within RStudio (Version 2024.12.1 + 563) to conduct a comprehensive bibliometric analysis. VOSviewer was used for bibliographic coupling data, while SPSS was used for correlation analysis. The Spearman correlation coefficient (*r*_*s*_) was used to evaluate the strength of relationships, categorized as high (*r*_*s*_ > 0.60), moderate (*r*_*s*_ > 0.30 ~ < 0.60), and weak (*r*_*s*_ < 0.30), with a significance level set at *P* ≤ 0.05.

## Results

Our search strategy for AI in ECG identified a total of 59,523 articles. We then reviewed the top 474 articles with the highest citation counts and were able to select the 50 most relevant and highest cited studies for our analysis. A detailed summary of all the 50 selected articles is presented in Table 1.

**Table 1:** 50 of the most cited articles on AI in ECG

Rank	Article title	Source title	Citations	Publication year	Citations density	Journal impact factor (journal citation reports 2023)
1	Kubios HRV—Heart rate variability analysis software	Computer Methods and Programs in Biomedicine	1870	2014	155.8	4.9
2	Cardiologist-level arrhythmia detection and classification in ambulatory electrocardiograms using a deep neural network	Nature Medicine	1598	2019	228.3	58.7
3	Real-Time Patient-Specific ECG Classification by 1-D Convolutional Neural Networks	IEEE Transactions on Biomedical Engineering	1167	2016	116.7	4.4
4	A wavelet-based ECG delineator: Evaluation on standard databases	IEEE Transactions on Biomedical Engineering	1122	2004	51	4.4
5	Automatic classification of heartbeats using ECG morphology and heartbeat interval features	IEEE Transactions on Biomedical Engineering	1040	2004	47.3	4.4
6	A deep convolutional neural network model to classify heartbeats	Computers in Biology and Medicine	838	2017	93.1	7
7	An artificial intelligence-enabled ECG algorithm for the identification of patients with atrial fibrillation during sinus rhythm: a retrospective analysis of outcome prediction	Lancet	812	2019	116	98.4
8	Dynamical model for generating synthetic electrocardiogram signals	IEEE Transactions on Biomedical Engineering	794	2003	34.5	4.4
9	Screening for cardiac contractile dysfunction using an artificial intelligence-enabled electrocardiogram	Nature Medicine	674	2019	96.3	58.7
10	Application of deep convolutional neural network for automated detection of myocardial infarction using ECG signals	Information Sciences	567	2017	63	0
11	A novel wavelet sequence based on deep bidirectional LSTM network model for ECG signal classification	Computers in Biology and Medicine	498	2018	62.3	7
12	Arrhythmia detection using deep convolutional neural network with long duration ECG signals	Computers in Biology and Medicine	495	2018	61.9	7
13	Automated detection of arrhythmias using different intervals of tachycardia ECG segments with convolutional neural network	Information Sciences	495	2017	55	0
14	ECG beat classification using PCA, LDA, ICA and Discrete Wavelet Transform	Biomedical Signal Processing and Control	488	2013	37.5	4.9
15	Automated diagnosis of arrhythmia using combination of CNN and LSTM techniques with variable length heart beats	Computers in Biology and Medicine	468	2018	58.5	7
16	ECG signal denoising and baseline wander correction based on the empirical mode decomposition	Computers in Biology and Medicine	467	2008	25.9	7
17	Automatic diagnosis of the 12-lead ECG using a deep neural network	Nature Communications	452	2020	75.3	14.7
18	PTB-XL, a large publicly available electrocardiography dataset	Scientific Data	436	2020	72.7	5.8
19	Deep learning approach for active classification of electrocardiogram signals	Information Sciences	416	2016	41.6	0
20	Clustering ECG complexes using Hermite functions and self-organizing maps	IEEE Transactions on Biomedical Engineering	405	2000	15.6	4.4
21	Cardiovascular Event Prediction by Machine Learning The Multi-Ethnic Study of Atherosclerosis	Circulation Research	387	2017	43	16.5
22	Optimal selection of wavelet basis function applied to ECG signal denoising	Digital Signal Processing	376	2006	18.8	2.9
23	Wavelet compression of ECG signals by the set partitioning in hierarchical trees algorithm	IEEE Transactions on Biomedical Engineering	360	2000	13.8	4.4
24	Classification of electrocardiogram signals with support vector machines and particle swarm optimization	IEEE Transactions on Information Technology in Biomedicine	358	2008	19.9	0
25	An Open Access Database for Evaluating the Algorithms of Electrocardiogram Rhythm and Morphology Abnormality Detection	Journal of Medical Imaging and Health Informatics	356	2018	44.5	0
26	Heartbeat Classification Using Morphological and Dynamic Features of ECG Signals	IEEE Transactions on Biomedical Engineering	349	2012	24.9	4.4
27	Support vector machine-based expert system for reliable heartbeat recognition	IEEE Transactions on Biomedical Engineering	349	2004	15.9	4.4
28	ECG beat recognition using fuzzy hybrid neural network	IEEE Transactions on Biomedical Engineering	348	2001	13.9	4.4
29	A Generic and Robust System for Automated Patient-Specific Classification of ECG Signals	IEEE Transactions on Biomedical Engineering	337	2009	21.1	4.4
30	The use of the Hilbert transform in ECG signal analysis	Computers in Biology and Medicine	337	2001	13.5	7
31	Automated diagnosis of Coronary Artery Disease affected patients using LDA, PCA, ICA and Discrete Wavelet Transform	Knowledge-Based Systems	334	2013	25.7	7.2
32	Automated processing of the single-lead electrocardiogram for the detection of obstructive sleep apnoea	IEEE Transactions on Biomedical Engineering	330	2003	14.4	4.4
33	A patient-adapting heartbeat classifier using ECG morphology and heartbeat interval features	IEEE Transactions on Biomedical Engineering	318	2006	15.9	4.4
34	ECG Arrhythmia Classification Using STFT-Based Spectrogram and Convolutional Neural Network	IEEE Access	317	2019	45.3	3.4
35	Real time electrocardiogram QRS detection using combined adaptive threshold	Biomedical Engineering Online	317	2004	14.4	2.9
36	ECG Classification Using Wavelet Packet Entropy and Random Forests	Entropy	312	2016	31.2	2.1
37	Arrhythmia recognition and classification using combined linear and nonlinear features of ECG signals	Computer Methods and Programs in Biomedicine	304	2016	30.4	4.9
38	Robust heart rate estimation from multiple asynchronous noisy sources using signal quality indices and a Kalman filter	Physiological Measurement	296	2008	16.4	2.3
39	Applying neural network analysis on heart rate variability data to assess driver fatigue	Expert Systems with Applications	288	2011	19.2	7.5
40	Multi-class Arrhythmia detection from 12-lead varied-length ECG using Attention-based Time-Incremental Convolutional Neural Network	Information Fusion	278	2020	46.3	14.7
41	Deep Convolutional Neural Networks and Learning ECG Features for Screening Paroxysmal Atrial Fibrillation Patients	IEEE Transactions on Systems Man Cybernetics-Systems	278	2018	34.8	8.6
42	Classification of myocardial infarction with multi-lead ECG signals and deep CNN	Pattern Recognition Letters	277	2019	39.6	3.9
43	A deep learning approach for real-time detection of atrial fibrillation	Expert Systems with Applications	275	2019	39.3	7.5
44	A deep learning approach for ECG-based heartbeat classification for arrhythmia detection	Future Generation Computer Systems-The International Journal of Escience	271	2018	33.9	6.2
45	A novel method for detecting R-peaks in electrocardiogram (ECG) signal	Biomedical Signal Processing and Control	270	2012	19.3	4.9
46	Block-based neural networks for personalized ECG signal classification	IEEE Transactions on Neural Networks	263	2007	13.8	10.2
47	Application of stacked convolutional and long short-term memory network for accurate identification of CAD ECG signals	Computers in Biology and Medicine	256	2018	32	7
48	ECG beat classifier designed by combined neural network model	Pattern Recognition	256	2005	12.2	7.5
49	Classification of 12-lead ECGs: the PhysioNet/Computing in Cardiology Challenge 2020	Physiological Measurement	251	2020	41.8	2.3
50	Support Vector Machines for Automated Recognition of Obstructive Sleep Apnea Syndrome From ECG Recordings	IEEE Transactions on Information Technology in Biomedicine	251	2009	14.8	0

### Publication timeline

**Fig. 1:**
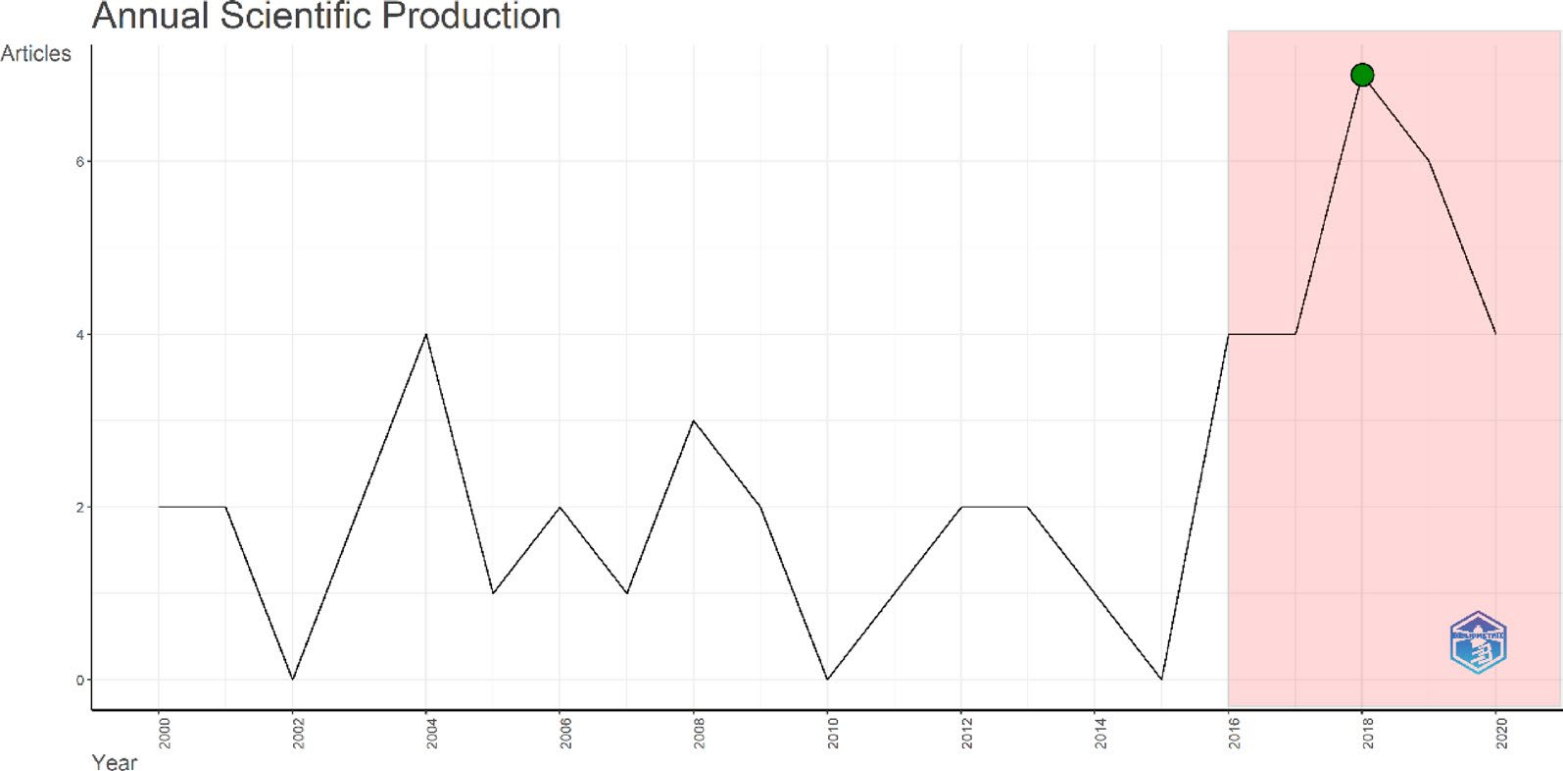
Annual scientific production timeline where red area presents the period of incremental increase in publications, while green dot represents the most publications in a year

The publication timeline of the included 50 articles on AI usage in ECG ranged from 2000 to 2020 and showed fluctuations. The first one and half decade of the publication timeline from 2000 to 2015 saw 25 (50%) publications in total, with 2004 having the highest (4) publications in this field. The publication timeline from 2016 till 2020 saw a huge increase in interest in this field with half (25) of the publications in these five years only. The highest number of publications in a year were 7, which were noted in 2018. The most cited article in our analysis, with 1870 citations, was published in 2014, while the least cited article, with 251 citations, was published in 2009. Furthermore, the results of Spearman correlation analysis revealed that there is a weak, non-significant correlation between publication year and number of citations (*r*_*s*_ = 0.002, *P* = 0.990), indicating that the year of publication does not influence how many citations an article receives in this dataset. Figure 1 illustrates the publication trend of the 50 most highly cited articles in this field.

### Citation analysis

**Fig. 2:**
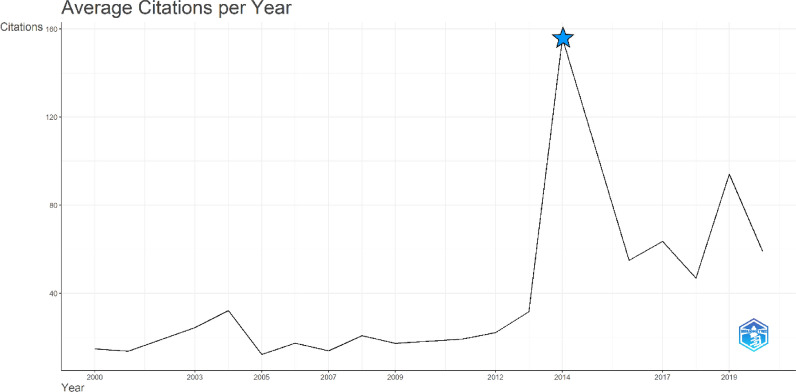
Average citations per year map where blue star represents the peak of citations

The 50 most cited articles in our dataset on AI in ECG gathered 24,401 citations in total till the date of our analysis. The highest citations of an article in our dataset were 1870, whereas the lowest citations an article had were 251. The mean citations of all the 50 articles in our dataset were 488.0 ± 340.7. The year 2014 saw the highest mean total citations per article of 1870.0 citations, and also the highest mean total citations per year of 155.8 citations. Figure 2 demonstrates the mean number of citations received per year by the 50 most cited articles.

The highest citations density recorded for an article was 228.3 published in 2019, while the lowest was 12.2 published in 2005. Moreover, Spearman correlation analysis was also conducted to examine the relationship between the impact factor of a journal and citation counts of the article in that journal. The findings showed a weak correlation (*rₛ* = 0.065, *P* = 0.655), indicating that there is no substantial evidence of a meaningful association between citations count and impact factor of a journal in this dataset.

### Journal and author analysis

**Fig. 3:**
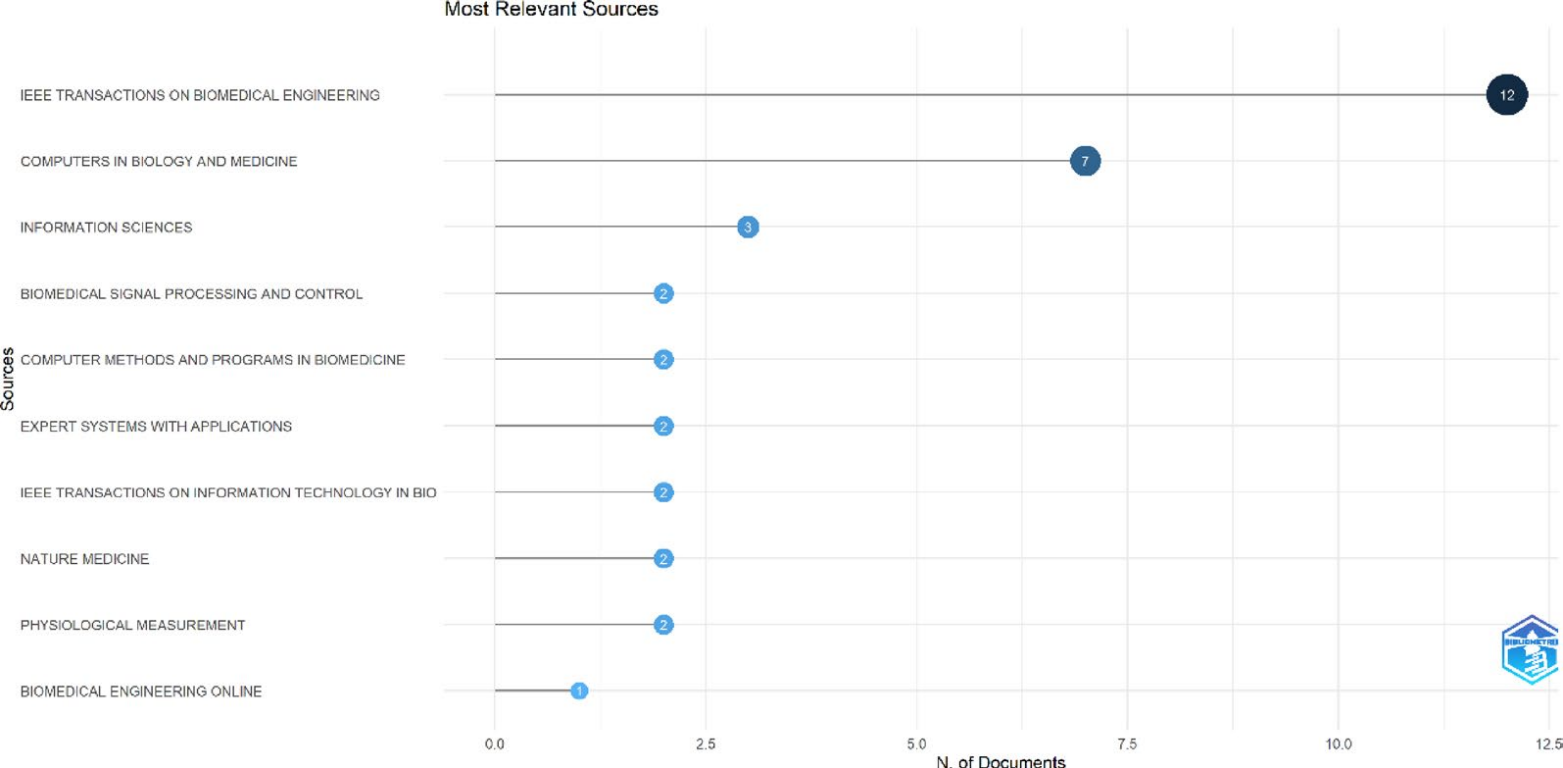
Journals with highest number of publications among the 50 articles

The 50 most cited articles on AI in ECG in our dataset were published across a total of 25 journals. The journal with most publications was ‘IEEE Transactions on Biomedical Engineering’ with 12 articles, followed by ‘Computers in Biology and Medicine’ journal with 7 publications. Other notable journals included ‘Biomedical Signal Processing and Control,’ ‘Nature Medicine,’ and ‘Expert Systems with Applications.’ The journal with highest impact factor according to journal citation reports 2023 was ‘Lancet’ with an impact factor of 98.4. Lancet was followed by ‘Nature Medicine’ with 2 articles and an impact factor of 58.7. Figure 3 lists the top ten journals in this field with most publications.

The authors which contributed the most in this field according to our dataset were 199 in total. Among these 199 authors, the author with most contribution and publications was ‘Professor Rajendra Acharya U’ with 9 publications and total citations count exceeding 4200. Professor Acharya was followed by ‘RS Tan’ with 5 publications and total citations count of 2334. Other notable authors include M Adam, Y Hagiwara, SL Oh, and JH Tan with 4 publications each. Table 2 shows the contributions of top ten authors in this field.

**Table 2:** Top 10 authors with most contributions in the field

Author	*H*-index	*M*-index	Total citations	Number of publications	Publication start year
Acharya UR	9	0.692	4218	9	2013
Tan RS	5	0.556	2334	5	2017
Adam M	4	0.444	2156	4	2017
Hagiwara Y	4	0.444	2156	4	2017
Oh SL	4	0.444	2129	4	2017
Tan JH	4	0.444	2156	4	2017
Clifford GD	3	0.13	1341	3	2003
DE Chazal P	3	0.13	1688	3	2003
Asirvatham SJ	2	0.286	1486	2	2019
Attia Zi	2	0.286	1486	2	2019

**Fig. 4:**
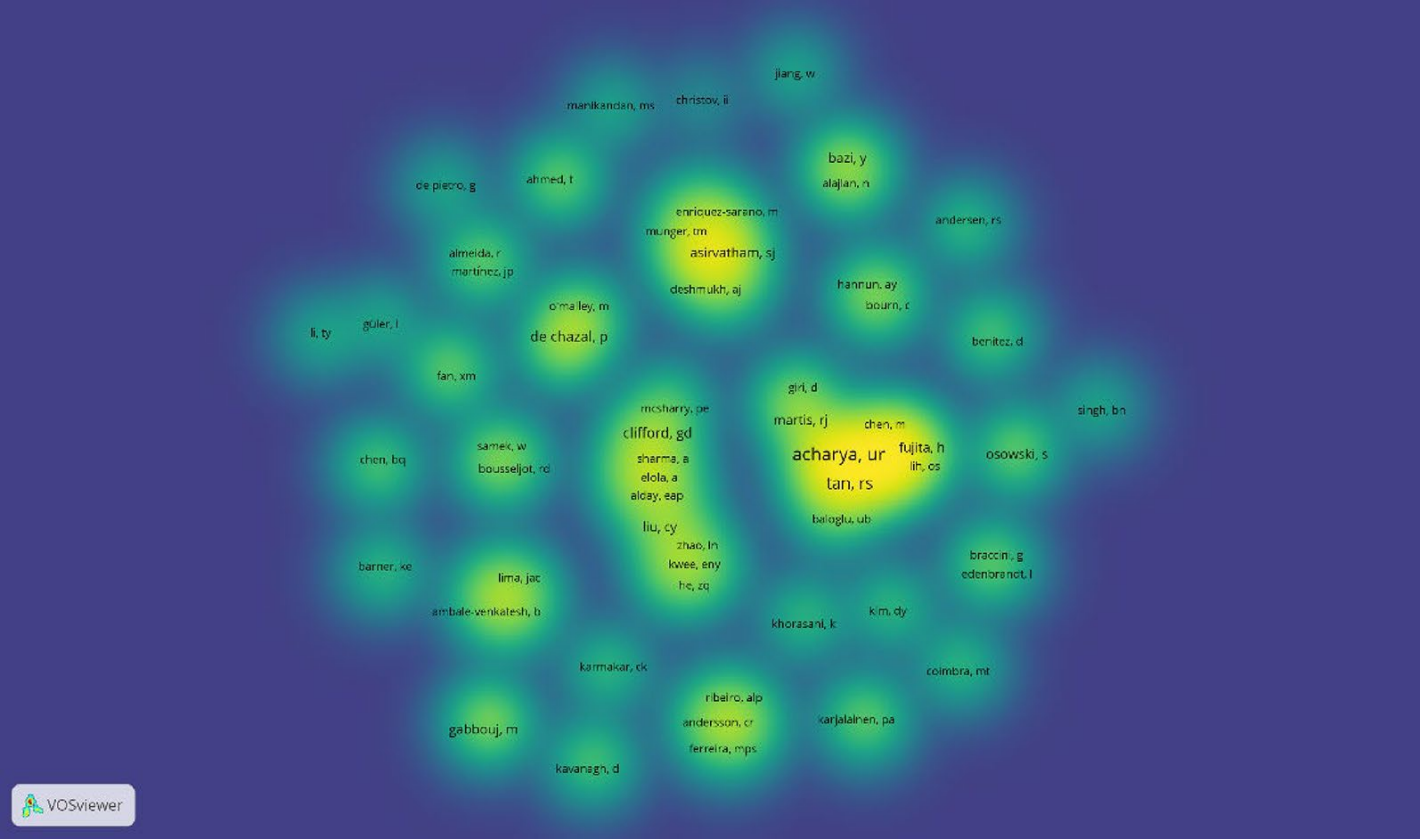
Visualization map of the most common keywords

Moreover, author collaborations within the field were analyzed, which are presented as density visualization map in Fig. 4. The collaboration network map offers a detailed representation of the scholarly connections and joint research efforts among authors. This analysis highlights the influence of individual authors and researchers and their collaborative networks in driving knowledge advancement and innovation within the field of AI in ECG.

### Analysis of countries and institutions

**Fig. 5:**
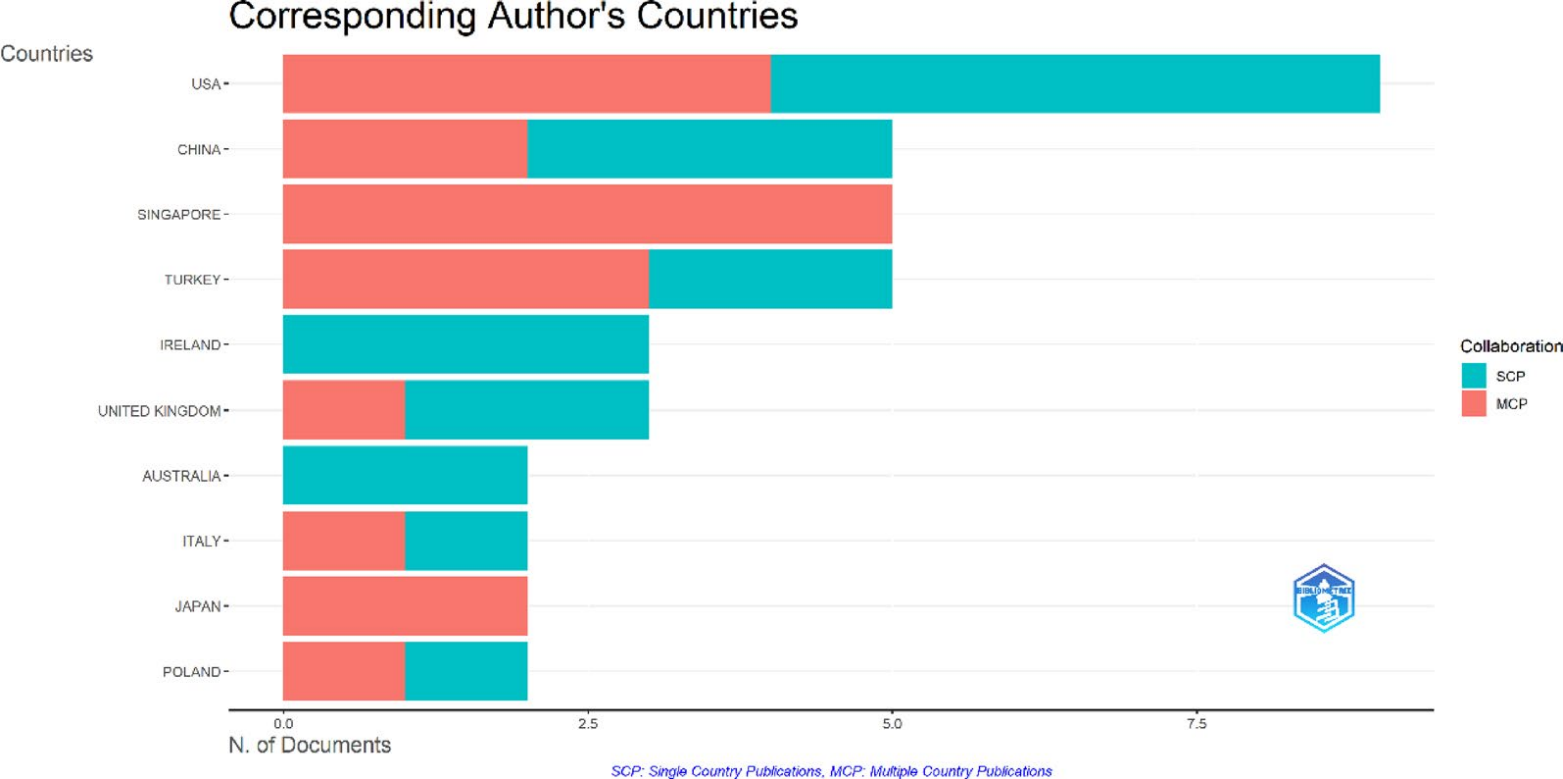
Top ten countries with the highest number of publications

A total of 21 countries contributed to this field according to our dataset. Among these 21 countries, the USA had the highest number of publications, with a total of nine articles. The USA was followed by China, Singapore, and Turkey with 5 publications each. Other notable countries were Ireland and the UK. List of top 10 countries with multiple country publication and single country publication is shown in Fig. 5.

**Fig. 6:**
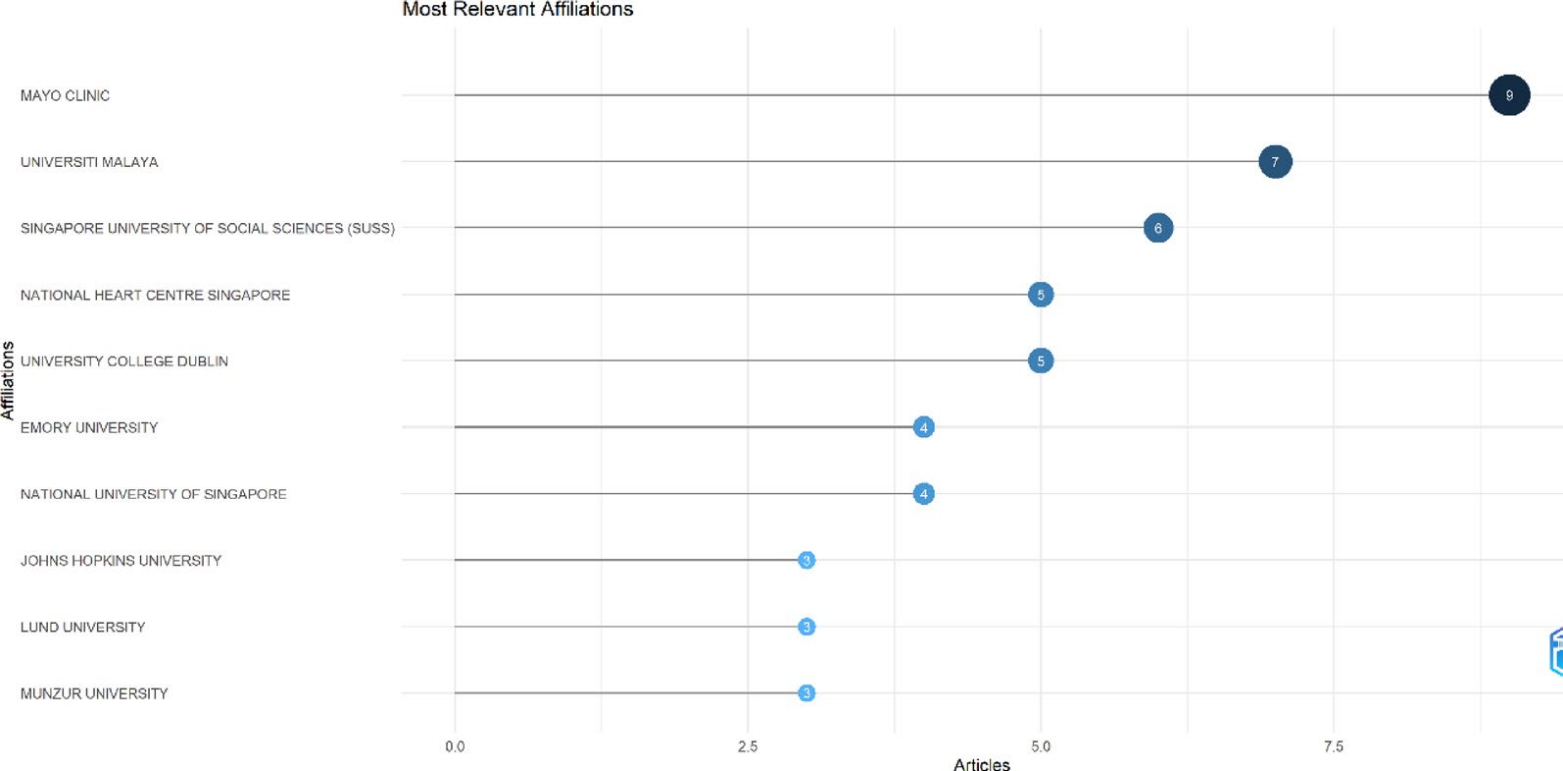
Institutions contributing the most publications in the field of AI in ECG

On the other hand, a total of 113 institutions contributed in this field, with Mayo clinic topping the list of publications. Mayo clinic contributed 9 articles and was followed by University of Malaya with 7 articles. Other notable institutions included Singapore University of Social Sciences, National Heart Centre Singapore, and University College Dublin. A detailed list of top ten institutions is shown in Fig. 6.

### Studies and keywords analysis

**Fig. 7:**
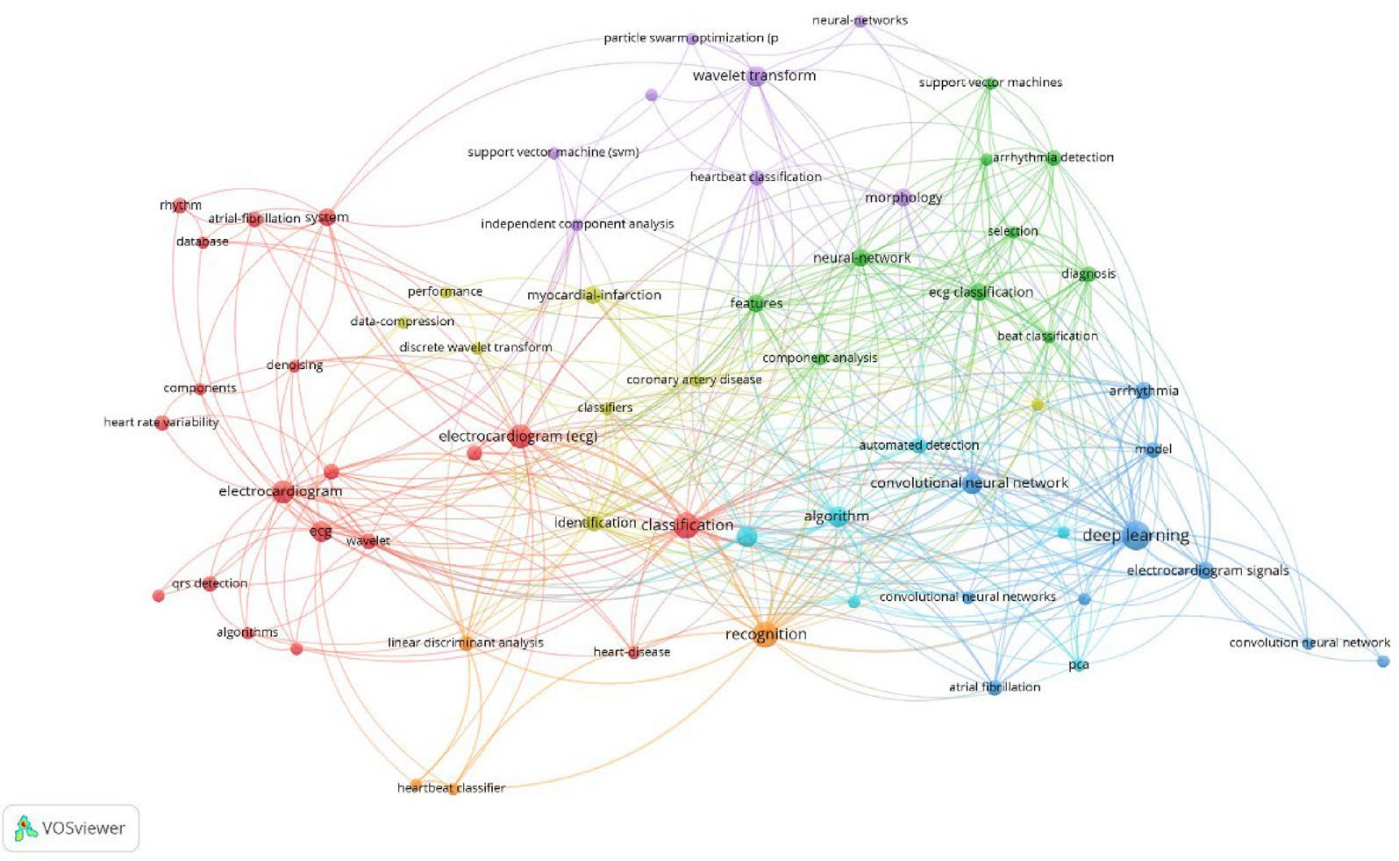
Network map of the most common keywords used in the 50 articles

**Fig. 8:**
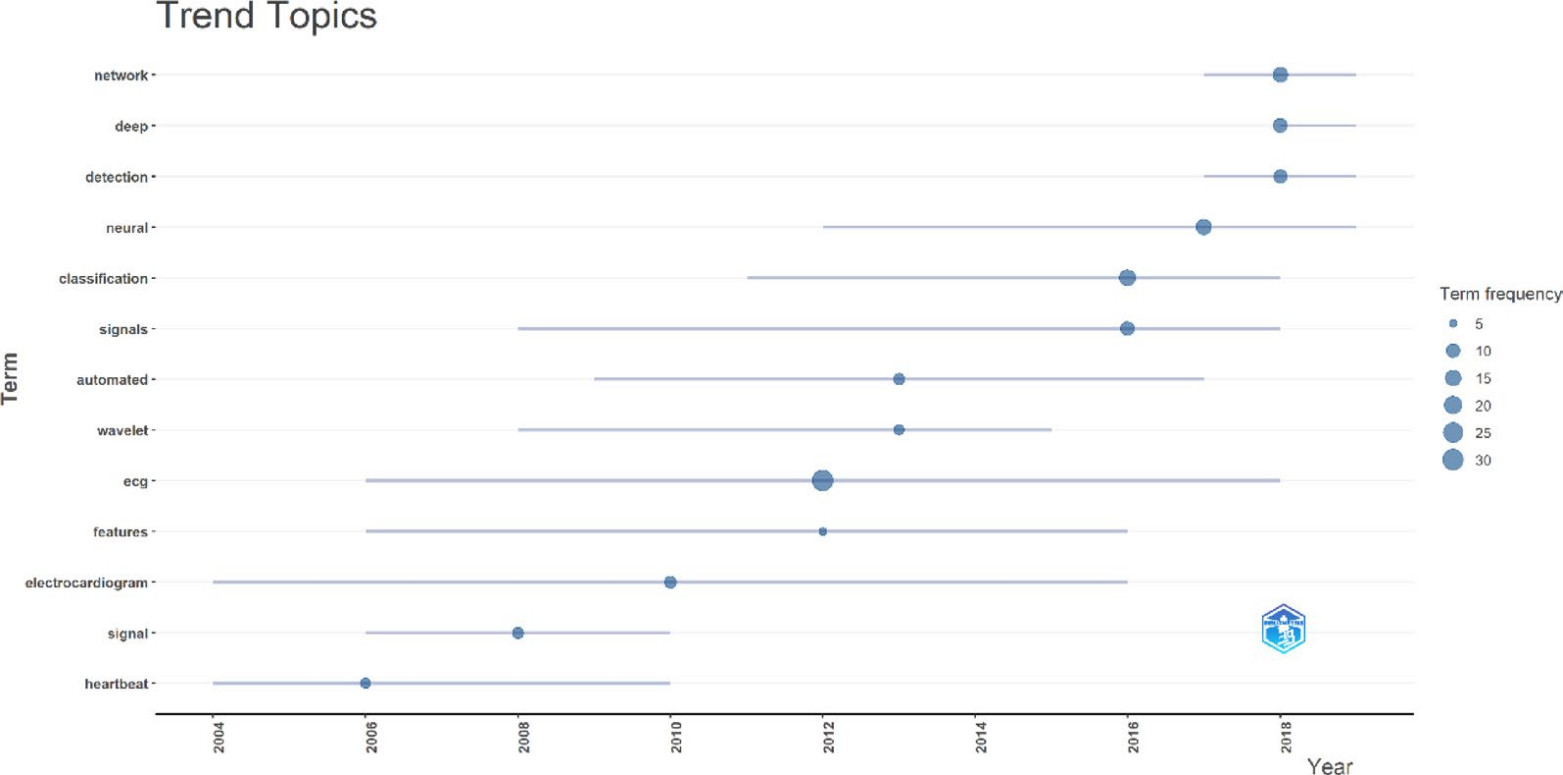
Top trending topics according to the most cited 50 articles

As the focus of this study was to highlight the most cited articles on AI use in ECG, so the included studies were mostly methodological or technical validation studies with LOE of 5. Other notable study types were cross-sectional studies, diagnostic accuracy studies, and cohort studies with LOE of 2b or more. Furthermore, the most common keywords used in the selected 50 articles included recognition, electrocardiogram, deep learning, classification, and neural network, among others. Figure 7 demonstrates the map of most common keywords. Moreover, a summary of trending topics is given in Fig. 8.

### Collaboration analysis

**Fig. 9:**
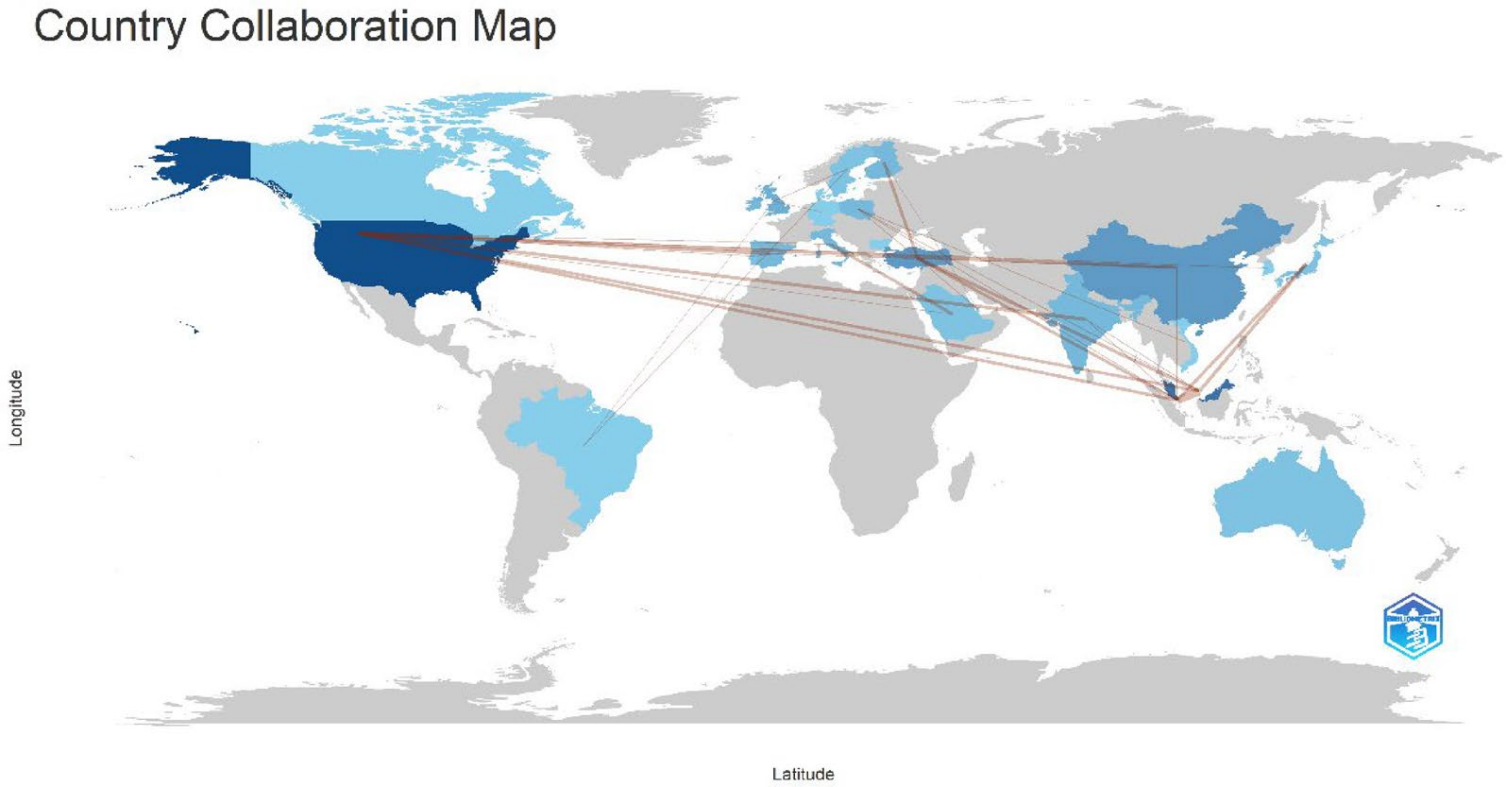
Country collaboration map of the countries

The global collaboration on research exploring AI usage in ECG demonstrated a complex and well-connected network. Notably, strong partnerships between Singapore and Malaysia, Italy and Saudi Arabia, Turkey and Finland, as well as the USA and China, underscore the shared commitment of these nations to advancing knowledge in this specialized field. A world collaboration map of the 50 most cited studies on AI in ECG is given in Fig. 9.

## Discussion

This bibliometric analysis aimed to highlight the 50 most cited articles on application of AI in ECG, and it revealed that this domain has vast existing literature and is continuously growing. Without any timeline restrictions, our analysis revealed that the field experienced ups and downs between the early 2000 s and 2010, but that interest in it saw a huge upward trend after 2016. The 5 years starting from 2016 had half (25/50) of the 50 most cited articles included in our analysis. These results can be associated with the increased recognition of AI in the field of ECG and other healthcare domains.

ECG is a diagnostic tool that is of significant importance and use of AI in it might further increase its diagnostic accuracy. The sharp increase in number of publications from 2016 aligns with the results of previous literature and shows the interest of researchers in AI usage in ECG [13]. Our analysis revealed no significant association between an article's publication year and its citation count, challenging the assumption that earlier publications accumulate more citations over time. Further analysis examining citations density demonstrated similar non-significant relation. Additionally, no significant relationship was observed between a journal's impact factor and the scholarly influence of individual articles as measured by citation frequency [14, 15]. These findings collectively suggest that temporal factors and the journal metrics may exert less deterministic influence on citation patterns than conventionally presumed within this research context.

The journals included in our analysis predominantly encompassed the fields of medicine and informatics, with prominent examples including IEEE Transactions on Biomedical Engineering, Computers in Biology and Medicine, Biomedical Signal Processing and Control, Nature Medicine, and Expert Systems with Applications. This distribution underscores the relevance of these journals as prospective platforms for disseminating research on AI applications in ECG. Furthermore, most of the included studies were methodological or validation studies with low LOE, highlighting the need of higher LOE studies in the field.

This is the first bibliometric analysis according to our knowledge which analyzed the 50 most cited articles on AI in ECG. Citation analysis serves as a principal methodology for identifying influential literature, as highly cited works reflect both their substantive disciplinary influence and foundational role in advancing research within a respective field [16]. Our analysis offers a systematic approach to streamline identification of seminal publications within the domain of AI in ECG, enabling researchers, scholars and clinicians to prioritize prominent works without requiring exhaustive review of existing literature. This analytical strategy may enhance efficiency in accessing high impact evidence while eliminating the necessity for comprehensive literature reviews, potentially supporting more targeted and evidence-based decision-making processes.

Although AI has seen many improvements in ECG and healthcare, there are still many areas that require more research. The integration of AI into routine clinical practice necessitates comprehensive training of healthcare professionals alongside systemic operational adjustments, representing a multifaceted and incremental transition; thus, advancing this field requires targeted research to address existing implementation barriers and optimize its adoption [17]. Future research should also prioritize establishing unified, interoperable platforms capable of integrating multi-modal ECG data into harmonized repositories. Building on existing resources, such infrastructures would accelerate AI innovation by enabling robust analysis of comprehensive datasets while promoting global collaboration through standardized data-sharing protocols [18].

We also want to highlight the limitations of our bibliometric analysis. First, our analysis relied on quantitative data, yielding overall insights into the studies within the field, rather than focusing on the clinical findings or implications of these studies. Second, we included only original articles and top 50 most cited articles, excluding review articles, systematic reviews, meta-analyses, conference papers, abstracts and even articles that satisfied the inclusion criteria but did not have enough citations; this may have resulted in the omission of some significant studies. Lastly, articles with high citations continue to get more citations in long run regardless of their relevance or integrity over the time known as the Matthew effect. To deal with this, we used citations density to check the impact of all the studies over time.

## Conclusion

This analysis highlighted the 50 most cited articles on AI usage in ECG that gathered over 24 thousand citations. The focus of researchers has shifted toward this domain in the last decade, especially after 2016. The USA, China, and several other Asian and European countries lead the field, reflecting their strong research infrastructures and commitment to advancing healthcare through technological innovation. The implementation of AI in diverse domains of ECG and cardiovascular system is anticipated to be a research priority in the forthcoming years.

## Data Availability

Data are provided within the manuscript or can be acquired from the corresponding author.

## Abbreviations

AI: Artificial intelligence
ECG: Electrocardiogram
WoSCC: Web of Science Core Collection
LOE: Level of evidence

## References

[1] Yu KH, Beam AL, Kohane IS (2018) Artificial intelligence in healthcare. Nat Biomed Eng 2(10):719–731. 10.1038/s41551-018-0305-z 31015651 10.1038/s41551-018-0305-z

[2] Chen RJ, Wang JJ, Williamson DFK et al (2023) Algorithmic fairness in artificial intelligence for medicine and healthcare. Nat Biomed Eng 7(6):719–742. 10.1038/s41551-023-01056-8 37380750 10.1038/s41551-023-01056-8 PMC10632090

[3] Koski E, Murphy J (2021) AI in healthcare. Stud Health Technol Inform 284:295–299. 10.3233/SHTI210726 34920529 10.3233/SHTI210726

[4] Jiang F, Jiang Y, Zhi H et al (2017) Artificial intelligence in healthcare: past, present and future. Stroke Vasc Neurol 2(4):230–243. 10.1136/svn-2017-000101 29507784 10.1136/svn-2017-000101 PMC5829945

[5] Siontis KC, Noseworthy PA, Attia ZI, Friedman PA (2021) Artificial intelligence-enhanced electrocardiography in cardiovascular disease management. Nat Rev Cardiol 18(7):465–478. 10.1038/s41569-020-00503-2 33526938 10.1038/s41569-020-00503-2 PMC7848866

[6] Kashou AH, May AM, Noseworthy PA (2020) Artificial intelligence-enabled ECG: a modern lens on an old technology. Curr Cardiol Rep 22(8):57. 10.1007/s11886-020-01317-x 32562154 10.1007/s11886-020-01317-x

[7] Breen CJ, Kelly GP, Kernohan WG (2022) ECG interpretation skill acquisition: a review of learning, teaching and assessment. J Electrocardiol 73:125–128. 10.1016/j.jelectrocard.2019.03.010 31005264 10.1016/j.jelectrocard.2019.03.010

[8] da Silva RMFL, de Souza MA (2021) Conduction diaorders: the value of surface ECG. Curr Cardiol Rev 17(2):173–181. 10.2174/1573403X16666200511090151 32392118 10.2174/1573403X16666200511090151 PMC8226204

[9] Baldassarre A, Mucci N, Padovan M et al (2020) The role of electrocardiography in occupational medicine, from Einthoven’s invention to the digital era of wearable devices. Int J Environ Res Public Health 17(14):4975. 10.3390/ijerph17144975 32664277 10.3390/ijerph17144975 PMC7400524

[10] Martínez-Sellés M, Marina-Breysse M (2023) Current and future use of artificial intelligence in electrocardiography. J Cardiovasc Dev Dis 10(4):175. 10.3390/jcdd10040175 37103054 10.3390/jcdd10040175 PMC10145690

[11] Powell KR, Peterson SR (2017) Coverage and quality: a comparison of web of science and scopus databases for reporting faculty nursing publication metrics. Nurs Outlook 65(5):572–578. 10.1016/j.outlook.2017.03.004 28377037 10.1016/j.outlook.2017.03.004

[12] Hassan MAU, Mushtaq S, Li T, Yang Z (2025) A bibliometric analysis of the 50 most cited articles about quality of life in patients with atrial fibrillation. Egypt Heart J EHJ Off Bull Egypt Soc Cardiol 77(1):21. 10.1186/s43044-025-00616-4 10.1186/s43044-025-00616-4 PMC11832831 39960571

[13] Sidik AI, Komarov RN, Gawusu S et al (2024) Application of artificial intelligence in cardiology: a bibliometric analysis. Cureus 16(8):e66925. 10.7759/cureus.66925 39280440 10.7759/cureus.66925 PMC11401640

[14] Kousha K, Thelwall M (2024) Factors associating with or predicting more cited or higher quality journal articles: an annual review of information science and technology (ARIST) paper. J Assoc Inf Sci Technol 75(3):215–244. 10.1002/asi.24810

[15] Chow NLY, Tateishi N, Goldhar A et al (2023) Does knowledge have a half-life? An observational study analyzing the use of older citations in medical and scientific publications. BMJ Open 13(5):e072374. 10.1136/bmjopen-2023-072374 37217270 10.1136/bmjopen-2023-072374 PMC10231019

[16] Shao B, Li X, Bian G (2021) A survey of research hotspots and frontier trends of recommendation systems from the perspective of knowledge graph. Expert Syst Appl 165:113764. 10.1016/j.eswa.2020.113764

[17] Aung YYM, Wong DCS, Ting DSW (2021) The promise of artificial intelligence: a review of the opportunities and challenges of artificial intelligence in healthcare. Br Med Bull 139(1):4–15. 10.1093/bmb/ldab016 34405854 10.1093/bmb/ldab016

[18] Feeny AK, Chung MK, Madabhushi A et al (2020) Artificial intelligence and machine learning in arrhythmias and cardiac electrophysiology. Circ Arrhythm Electrophysiol 13(8):e007952. 10.1161/CIRCEP.119.007952 32628863 10.1161/CIRCEP.119.007952 PMC7808396

